# [Retracted] MicroRNA‑362 is downregulated in cervical cancer and inhibits cell proliferation, migration and invasion by directly targeting SIX1

**DOI:** 10.3892/or.2024.8713

**Published:** 2024-02-06

**Authors:** Can Shi, Zhenyu Zhang

Oncol Rep 37: 501–509, 2017; DOI: 10.3892/or.2016.5242

Following the publication of the above article, a concerned reader drew to the Editor's attention that the Transwell cell migration and invasion assay data featured in Fig. 2C were strikingly similar to data appearing in different form in other articles written by different authors at different research institutes that had either already been published elsewhere prior to the submission of this paper to *Oncology Reports*, or were under consideration for publication at around the same time (some of which have been retracted). Furthermore, a number of overlapping data panels were identified comparing the migration and invasion assay data in Figs. 5C and 6C, such that data which were intended to show the results from differently performed experiments appeared to have been derived from the same original source(s).

In view of the fact that certain of these data had already apparently been published previously, and given the lack of rigour on the part of the authors in assembling the data in Figs. 5 and 6, the Editor of *Oncology Reports* has decided that this paper should be retracted from the Journal. The authors were asked for an explanation to account for these concerns, but the Editorial Office did not receive a satisfactory reply. The Editor apologizes to the readership for any inconvenience caused.

